# Positive psychology interventions in in-patients with depression: influences of comorbidity and subjective evaluation of the training programme

**DOI:** 10.1192/bjo.2021.65

**Published:** 2021-06-03

**Authors:** Antje Stemmler, Regina Staehle, Tina Heinemann, Matthias Bender, Juergen Hennig

**Affiliations:** Department of Personality and Biological Psychology, Faculty of Psychology and Sports Sciences, Justus Liebig University of Giessen, Germany; Department of Psychology, University of Kassel, Germany; Department of Psychology, University of Kassel, Germany; Vitos Hospital for Psychiatry and Psychotherapy, Germany; Department of Personality and Biological Psychology, Faculty of Psychology and Sports Sciences, Justus Liebig University of Giessen, Germany; and Center for Psychobiology and Behavioral Medicine, Justus Liebig University of Giessen, Germany

**Keywords:** Comorbidity, group psychotherapy, in-patient treatment, positive psychology, depression

## Abstract

**Background:**

Studies on positive psychology interventions (PPIs) have frequently demonstrated benefits for healthy participants and patients. However, effect sizes are moderate, and underlying inter-individual differences in responses were rarely investigated.

**Aims:**

We investigated whether severity of depression and subjective evaluation of PPIs are relevant sources of variance in this respect.

**Method:**

A 4-week group PPI programme (one 45-min session per week) was offered to 38 in-patients with depression. The control group (*n* = 38) was carefully matched and received treatment as usual. In the PPI group, emotional states were recorded before and after each session (responsiveness). Beck Depression Inventory-II scores at hospital admission and discharge were used to evaluate clinical effectiveness. The number of comorbidities (as an indicator of severity of disease) and patients’ evaluations of the PPI sessions were used as additional independent factors for overall treatment outcome.

**Results:**

The PPI induced a highly significant improvement in positive emotional state and decrease in negative emotional state, indicating responsiveness. Moreover, positive affectivity increased from week to week only in patients with a low number of comorbidities (indicating effectiveness). With respect to overall treatment outcome (Beck Depression Inventory-II scores), positive attitude toward the PPI resulted in the largest improvement.

**Conclusions:**

The results partly explain the variance in the effectiveness of PPIs. Moreover, they support the idea of personalised psychotherapy, and may inform discussion on whether patients with depression should be included in PPIs. However, additional individual characteristics should increase knowledge about individual predictors for effectiveness.

## Positive psychology

It is well documented that humans suffer from a negativity bias or, as Baumeister et al put it, ‘bad is stronger than good’.^1^ What is true for human perception is also true for the history of psychological research, which traditionally focused on pathology and mental illness instead of strength, growth and well-being.^2^ In the late 1990 s, however, Martin Seligman noticed this imbalance. His findings, combined with much enthusiasm to spread them throughout the scientific community, established the field of positive psychology.

Positive psychology is ‘the study of the conditions and processes that contribute to flourishing or optimal functioning of people, groups, and institutions’.^3^ One of the primary goals of positive psychological research is the development and evaluation of positive psychology interventions (PPIs), which are intentional activities or treatment methods that help to reach flourishing and optimal functioning. Successful PPIs, in other words, cultivate positive feelings, behaviours or thoughts,^4^ and include examples such as gratitude exercises, practices using personal strengths, and forgiveness.

## Previous research and goal of the study

In 2005, Seligman et al investigated the effects of five self-administered PPIs in a sample of participants who visited the website of Seligman's 2002 book ‘*Authentic Happiness*’.^5^ Participants (*N* = 411) had mild depression, according to Center for Epidemiologic Studies Depression Scale (CES-D) scores measured at baseline. Two interventions (the ‘three good things’ writing exercise and the signature strength exercise) were associated with increased happiness, even after 6 months. A study in 2006 tested the effects of PPIs in an individual therapy setting, and also found positive results.^6^ Participants included 41 patients with major depressive disorder ranging from mild to moderate, as determined by the Beck Depression Inventory-II (BDI-II; scores 10–24).^7^ Results showed that participants who received PPIs showed more symptomatic improvement and a larger increase in happiness, compared with participants who received treatment as usual (TAU; both with and without antidepressant medication).

There is, thus, evidence of PPI effectiveness in clinical patients. For therapeutic applications, however, group interventions are particularly interesting because of their economic advantages and the utilisation of helpful group synergies. Other studies have focused on PPI in a group therapy context.

Chaves et al tested PPIs in a ten-session group therapy programme of 32 women diagnosed with depression or dysthymia.^8^ Results show that PPIs reduced psychopathology and increased positive functioning, with similar effect sizes as a control protocol of cognitive–behavioural therapy (CBT). Moreover, even in patients with severe depression, there was no difference in efficacy between both treatment conditions. Another group intervention study also compared PPIs with CBT and found similar results.^9^ All 18 participants were diagnosed with major depressive disorder (mild, moderate or severe), had no history of psychotherapy and no comorbidities. Both interventions were effective in decreasing depression, but the PPI was more effective in increasing happiness than the CBT. Furthermore, Furchtlehner et al investigated the efficacy of a 14-week PPI group programme compared with a CBT group programme.^10^ They included a larger sample of 92 patients with mild-to-moderate major depressive disorder, with dysthymia or double depression. Both treatments reduced depressive symptoms significantly, but there was a significantly larger reduction in BDI-II score in the PPI group than the CBT group. BDI-II scores were further significantly reduced at 6 months after treatment, for both interventions.

A recent meta-analysis documents the overall evidence of the general effectiveness of PPIs.^11^ It includes 347 studies published in peer-reviewed academic journals in 41 countries. The final cohort comprised 72 356 participants (70.3% non-clinical patients, 13.5% had a physical disorder and 16.4% had a mental health disorder). The study shows that PPIs have significant effects in the small-to-medium range: they enhance strengths, well-being and quality of life (*g* = 0.46, *g* = 0.39 and *g* = 0.48, respectively); and alleviate depression, anxiety and stress (*g* = −0.39, *g* = −0.62 and *g* = −58, respectively). After removing low-quality studies (nearly 50%), changes in effect sizes were small and not significant.

However, studies have rarely investigated patients with severe depression or those receiving in-patient treatment. Furthermore, previous studies mostly did not consider comorbidities, which are usually of crucial importance in the treatment of depression.^12–14^ Additionally, it can be expected that the effectiveness of a PPI depends on the evaluation of or attitude toward PPI. It should therefore be investigated whether increases in well-being and reduction in clinical depression are more pronounced in patients who consider PPI to be helpful and beneficial.

The present study seeks to close these gaps by focusing on the effects of PPI in in-patients with depression, while controlling for comorbidities. We hypothesise that the positive effects of PPIs generalise to a sample of in-patients with severe depression, especially in those with positive experiences after PPI. However, we also expect that the beneficial effects of PPI will be diluted in relation to an increasing number of comorbidities.

## Method

### Sample

Patients were recruited in the affective disorder in-patient units of the Vitos Hospital for Psychiatry and Psychotherapy in Kassel, Germany. Between March and September of 2019, all admitted patients were invited to participate if they met the diagnostic criteria for a depressive episode or recurrent depressive disorder according to ICD-10 criteria (codes F32 or F33, respectively).^15^ We excluded patients who suffered from schizophrenic disorders or bipolar affective disorders because psychotherapeutic interventions play a smaller role in the treatment of these patients. Further excluded were patients who were not able to attend group therapy sessions. All participants were informed about the study and signed an informed consent form; they did not receive any compensation. The study was approved by the local ethics committees of the Department of Psychology (2018-0017) and the Department of Medicine (AZ 28/19) at the Justus Liebig University of Giessen.

Participants were randomly assigned to one of two treatment conditions by proxy of the ward to which they were admitted. Ward one received TAU, whereas ward two additionally received the PPI. Since the assignment to the wards was solely based on current capacity, it can be considered random. The wards both specialise in treating affective disorders and are led by the same head physician. This mapping of ward to treatment condition served practical purposes, such as avoiding possible confusion among patients over selective treatment opportunities.

All patients were assigned to a complex therapy programme that included pharmacotherapy, one 50-min individual CBT session per week, ergotherapy, kinesitherapy and therapeutic group treatments based on individual needs. Additionally, in the PPI condition, participants took part in a weekly 45-min group intervention for four consecutive weeks.

In total, 111 patients took part in the study, 68 of whom were female (61.3%). Patients were, on average, 44.31 years old (s.d. 14.43). A total of 57 patients were assigned to the TAU condition, 36 of whom were female (63.2%); and 54 were assigned to the PPI condition, 32 of whom were female (59.3%). The average number of diagnoses, as assessed with the German version of the Structured Clinical Interview for DSM-IV (SCID; see below), was 2.45 (s.d. 1.37).

After the first wave, 38 patients completely finished the PPI training without any missing data. In each condition there was one patient (2.6%) who did not receive psychiatric medication. To these patients, we carefully matched an equal number of patients from the TAU condition. Matching was based on gender, age, number of comorbidities, number of previous in-patient treatments and BDI-II classifications (number of patients with mild, moderate and severe depression in each group; see Table 1).

**Table 1:** Matched samples according to matching criteria and diagnoses for each group

Criterion	PPI (*n* = 38)	TAU (*n* = 38)	Statistic	*P-*value
Age, years, mean (s.d.)	43.31 ± 16.32	45.02 ± 13.26	*t* = 0.501	Not significant (0.62)
Number of previous in-patient treatments, mean (s.d.)	1.79 ± 2.24	2.29 ± 2.70	*t* = 0.87	Not significant (0.39)
Number of comorbidities, mean (s.d.)	1.26 ± 1.22	1.45 ± 1.26	*t* = 0.65	Not significant (0.52)
Gender, *n*	Female: 20; male: 18	Female: 20; male: 18	*χ*^2^ = 0	Not significant (1)
BDI-II classification at admission, *n*			*χ*^2^ = 1.47	Not significant (0.48)
Mild	2	4		
Moderate	12	8		
Severe	24	25		
ICD-10 diagnosis, *n*				
F10.1	1	1		
F10.20		1		
F11.20		1		
F12.1	1			
F12.202		1		
F15.1	1			
F15.5	1			
F32.0	1			
F32.1	1			
F32.2	8	4		
F33.1	6	3		
F33.2	21	28		
F33.3	2	2		
F34.1	9	15		
F40.0	3	3		
F40.01	4	5		
F40.1	13	12		
F40.2	4	4		
F41.0	2	6		
F41.1	1			
F42.1		1		
F43.1	6	5		
F44.6		1		
F45.2	1			
F50.3	1			
Total	87	93		

PPI, positive psychology intervention; TAU, treatment as usual; BDI-II, Beck Depression Inventory.

### Diagnostic instruments

We used the German versions of three diagnostic instruments – the SCID-I, BDI-II and Positive and Negative Affect Schedule (PANAS) – for diagnoses, estimates of the severity of depression and for measuring changes in emotional states after PPIs, respectively.

The SCID (in German: *Strukturiertes Klinisches Interview für DSM-IV* (SKID)) is a semi-structured, standardised clinical interview for adults, yielding diagnoses according to the DSM-IV.^16^ The SCID comprises two separate parts. The SCID-I allows for the diagnosis of several disorder groups: affective disorders, psychotic disorders, mental and behavioural disorders due to psychoactive substance use, anxiety disorders, somatoform disorders, eating disorders and adaption disorders. The SCID-II is used to diagnose personality disorders. In our study, we did not use the SCID-II because personality disorder diagnostics should not be done during mental crises, which in-patients are experiencing.

The SCID is a complex diagnostic instrument that can be considered as the gold standard of diagnostic interviewing. According to its manual, it takes about 100 min to complete and should only be administered by psychologists or psychiatrists who know the classification manual, have in-clinic psychiatric experience, and complete a 2-day training programme. In our study, the SCID was administered by psychologists working at the Vitos Hospital for Psychiatry and Psychotherapy (A.S., R.S. and T.H.). Values for test-retest reliability and interrater reliability are fair to excellent.^17,18^ Although recommended, the SCID is not used in many studies because of its time-consuming application.

Our second diagnostic instrument, the German version of the revised BDI-II,^19^ is a standard self-report questionnaire that captures the severity of depression. It comprises 21 items, each with four statements that refer to symptoms of depression. Each item contains statements in order of increasing severity. For two items (changes in sleep patterns and changes in appetite), seven statements reflect both negative and positive deviations. In any case, participants select the one statement that best describes how they have felt during the past 2 weeks. Each item then receives a score between 0 and 3, yielding a scale value between 0 and 63. Total values from 0 to 9 indicate no or minimal depression, values from 10 to 18 indicate mild depression, values from 19 to 29 indicate moderate depression and values above 29 indicate severe depression.^19^ The BDI-II has high reliability, with Cronbach's alpha values between *α* = 0.89 and *α* = 0.93.^19^

The German version of the PANAS^20^ assesses emotional states. The PANAS comprises a list of 20 adjectives and is based on self-reports with two underlying independent factors: positive affect and negative affect. Adjectives that are used to describe positive affect include enthusiastic, active and attentive. Examples for negative include irritability, nervousness and anxiety. The intensity is assessed on a five-point Likert scale, with 1 being very slightly or not at all, 2 being a little, 3 being moderately, 4 being quite a bit and 5 being extremely. PANAS has high psychometric quality: its internal consistency (Cronbach's alpha) is at a high *α* = 0.85 for positive affect and *α* = 0.86 for negative affect, and there is evidence for differential internal validity and external validity.^20^ Finally, we added a short questionnaire for the evaluation of the PPI sessions, and applied it to patients immediately after each session. A total number of six items should be answered on whether ‘the session was interesting’, ‘the session was exciting’, ‘the session was fun’, ‘I would like to continue with these sessions’, ‘I can recommend participating in these sessions’ and ‘the session was good for me’. Answers can range between not at all (1) and totally (5).

### Study design

In this study, we used a two-factor between-groups design. One independent variable was the condition (PPI or TAU). In the PPI condition, participants received a PPI in addition to standard treatment. TAU served as a control group. A second independent variable with two levels was the number of diagnoses assessed with the SCID interview. We used the BDI-II to measure the change in depressive symptoms pre- and post-treatment, and the PANAS to measure the change in affectivity in the context of the PPI sessions (before and after each session) as dependent variables.

### Procedure

Before any treatment, all patients were subjected to a diagnostic interview (SCID-I) and completed a set of questionnaires (including the BDI-II). Before hospital discharge, patients completed the same set of questionnaires.

The PPI group treatment programme consisted of four modules. Each week, we offered two sessions for one of the modules, giving patients a chance to choose the one that fit their schedule best. We designed the modules to be independent of each other so that patients could start the PPI programme at any time. This guaranteed that all patients assigned to the PPI group could start the programme right after admission to the hospital (i.e. within their first week).

Each module focused on one exercise. The exercises we used were selected based on two criteria: evidence of effectiveness and feasibility in the in-patient setting. Before and after each session, we asked participants to complete the PANAS questionnaire on a tablet computer. The group sessions had no more than eight participants and were conducted by one of three instructors. To keep differences between the instructors to a minimum, they were thoroughly trained and followed a standardised treatment manual, which defined content and wording of instructions.

Module one focused on psychoeducation. The discussion centred around our negativity bias and its implications for goal setting, as well as the goal of the intervention. At the end of the group session, participants were introduced to the take-home exercise ‘three good things in life’.^6^ It involves writing down ‘three things that went well each day and their causes every night for one week’.^6^ Seligman et al^5^ tested this exercise with visitors of Seligman's website and found an increase in happiness alongside a decrease in depressive symptoms after 6 months. This finding is in line with recent studies such as that by Gander et al.^21^ For our participants, we used a simplified version of that task: instead of three, we asked them to write down two things.

Module two was about identifying and fostering strengths, inspired by previous studies that showed the efficacy of strengths-based interventions.^6,21,22^ It began by collecting previous knowledge of strengths and discussing the connection with well-being. A previously published model for character strengths and virtues was introduced.^23^ Participants had to choose at least three strengths that they attributed to themselves. Additionally, they had to reflect on specific situations in which their character strength became apparent. They were also encouraged to apply their strengths in new situations. For this purpose, reasonable goals and specific behaviours in everyday life were developed. The take-home exercise had two parts. In the first part, participants had to think of an adverse event that they had overcome successfully. They had to describe the event, their behaviour and the strengths that appeared. In the second part, participants had to ask a trusted person for feedback on a situation in which one of their strengths emerged.

Module three focused on active constructive responding (ACR), defined as ‘an active constructive response is one where you react in a visibly positive and enthusiastic way to good news from someone else’.^7^ ACR is positively related to relationship well-being, and helps to build social resources by fostering positive social interactions.^24^ Seligman et al implemented ACR as part of a 6-week PPI group treatment and showed a significant reduction in depressive symptoms and improved life satisfaction.^6^ In our PPI programme, we introduced ACR by using role-play to contrast different response styles to positive events reported by others. Participants had to observe the behavioural differences between the different response styles, and the results were captured on a flipchart. The benefits of ACR were discussed in the group. The take-home exercise of module three was to reflect on the strengths of two close people. In addition, participants were instructed to give these people feedback in an active-constructive way, and take note of their experiences.

Module four was about growth through adversities (‘one door closes, another door opens’).^25^ In this activity, participants write (daily, for one week) about a negative event that had unforeseen positive consequences. Again, we used a modified version of the task. The group session started with the old Chinese parable of the farmer and his horse, which the instructor read to the participants. (In the parable, a Chinese farmer has to cope with a number of adversities but later realises that they also have positive effects. For instance, one day his horse escapes, which is a major loss to him. However, after some time, it comes back in a team of other horses.) Then the message of the story was discussed. We highlighted the importance of valuations and the active role in dealing with negative events with the aim of broadening participants’ perspectives. The take-home exercise was to think of two small negative events (failure, rejection or loss) and write down the positive aspects of the experiences. For further assistance, participants received worksheets with questions that could help them detect positive aspects of negative events.

### Data analysis

All data analyses and statistical tests were performed in SPSS, version 25 for MacOS. The first independent variable was operationalised as a binary factor, reflecting patients’ treatment condition (PPI or TAU). The second independent variable, the number of diagnoses, was measured as the sum of individual diagnoses that emerged from the SCID-I interview described above. Because of its skewed distribution, this measure was reduced to a binary factor with the levels low, representing one or two diagnoses, and high, representing three and more diagnoses; this transformation to a binary variable yielded two groups of approximately the same size. Because of our careful matching (see above), an equal distribution of 23 patients with a low number of diagnoses and 15 with a high number of diagnoses can be found in the PPI and TAU group.

Since all of the items concerning ratings toward PPI were highly significantly correlated (*r* = 0.45–0.69), we computed a mean score across all items and divided them into two groups by median dichotomisation. This resulted in three groups: no experience with PPI (TAU, *n* = 38), a moderately positive attitude toward PPI (*n* = 18) and a highly positive attitude toward PPI (*n* = 20).

The effect of the PPI on emotional states (responsiveness) was calculated by a two-factor (group and comorbidity, each with two levels) repeated measures ANOVA, with two within-group factors (session with four levels and pre–post with two levels, respectively). PPI effectiveness was tested by repeated measures ANOVA, with comorbidity as the between-patient factor, the first session as a covariate and one within-group factor (emotionality before sessions 2–4). Finally, changes in BDI-II scores according to PPI versus TAU and experience of PPI were calculated with two factors (group with two levels and experience with three levels: no experience or TAU, moderately positive attitude toward PPI, highly positive attitude toward PPI). Experience was related to changes in BDI-II scores after treatment, using repeated measures ANOVA (factor experience with three levels; admission versus discharge). Effect sizes (*η*^2^) are given for all analyses.

## Results

**Fig. 1:**
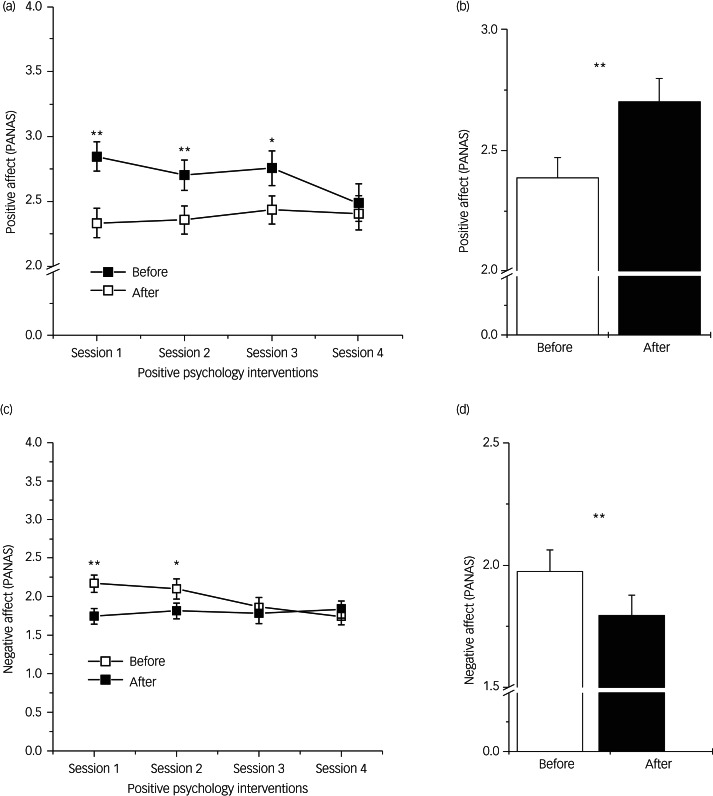
Positive and Negative Emotionality prior to and after PPI. (a) Differences in Positive Emotionality across all PPI sessions. (b) Main effect of changes in Positive Emotionality (mean levels across all sessions). (c) Differences in Negative Emotionality across all PPI sessions. (d) Main effects of changes in Negative Emotionality (mean levels across all sessions). All data are expressed as means ± SEM. PANAS, Positive and Negative Affect Schedule. *P < 0.05, **P < 0.01.

According to our hypotheses, we first investigated the responsiveness to PPI by using the scale values of the PANAS in a repeated-measure design (Fig. 1).

Repeated measures ANOVA clearly indicate a highly significant main effect for the comparison of emotionality before and after the intervention (positive affect: *F* = 24.84, d.f. = 1.35, *P* < 0.01, *η*^2^ = 0.41; negative affect: *F* = 18.01, d.f. = 1.35, *P* < 0.01, *η*^2^ = 0.34; see Fig. 1(b)–(d)). Moreover, a significant interaction can be demonstrated between both repeated measure factors (session and pre–post) for positive emotionality (*F* = 3.26, d.f. = 3.33, *P* < 0.05, *η*^2^ = 0.23) and negative emotionality (*F* = 6.48, d.f. = 3.33, *P* < 0.01, *η*^2^ = 0.37; see Fig. 1(a)–(c)). *Post hoc* tests (*t*-tests for dependent samples) demonstrate that the effects of PPI become lower and non-significant in the last session (positive emotionality) and the final two sessions (negative emotionality), respectively. These results demonstrate that PPI is, in fact, efficient (see main effects), but becomes less beneficial at the end of the programme. To summarise at this point, it can be concluded that the responsiveness to PPI is not unaffected by the number of sessions. Since patients could enter the PPI programme at any point in time, this reduction of effectiveness does not relate to the content of the sessions.

**Fig. 2:**
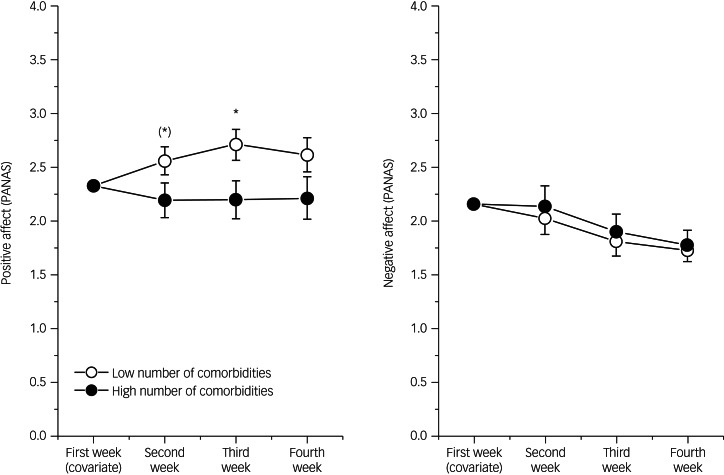
Means and SEM for Positive Emotionality prior to every PPI within four consecutive weeks in patients with a high vs. low number of diagnoses (comorbidities). Whereas both groups did not differ with respect to Negative Emotionality (right), only those patients with fewer comorbidities improved significantly (increases in Positive Emotionality, left), especially after two and three weeks of treatment. PANAS, Positive and Negative Affect Schedule. (*)*P* < 0.08, *P < 0.05.

Our second hypothesis related to the question of whether the number of comorbidities affects the effectivity of the PPI. We expected that a higher number of comorbidities would result in lower week-to-week changes in emotional states, controlling for unspecific differences at the beginning of the training. Results for positive and negative emotionality are depicted in Fig. 2.

PPI increased positive emotionality from week to week only in patients with a low number of diagnoses (fewer comorbidities) (Fig. 2). Since this difference between the two groups occurred in every session, the interaction between session and group did not become significant (*F* = 0.25, d.f. = 2.70, *P-*value was not significant). In contrast, both groups differed systematically, leading to a significant main effect (*F* = 5.14, d.f. = 1, *P* < 0.05, *η*^2^ = 0.13). This clearly indicates that patients with a lower number of comorbidities benefitted more from PPI. With respect to negative emotionality, no differences could be observed between both groups.

**Fig. 3:**
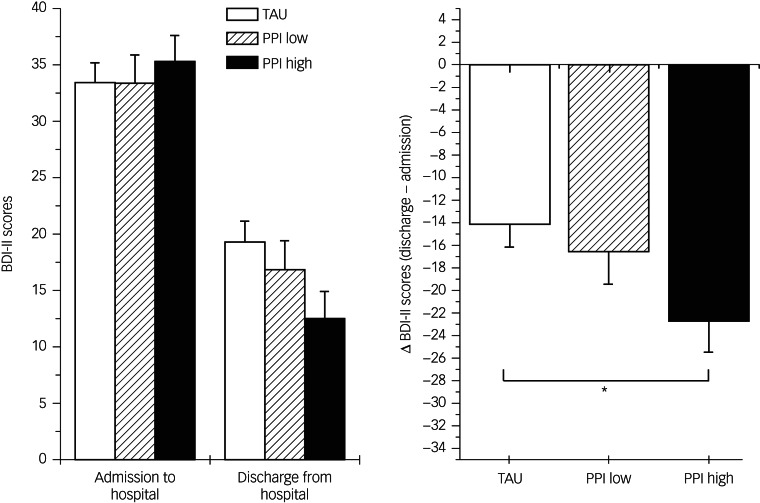
Means and SEM of BDI-II scores before and after treatment in patients without experiences in PPI, patients who valued very much (PPI high) and those who did not (PPI low) with pre and post values (left side) and differences scores (post minus pre, right). BDI-II, Beck Depression Inventory-II; PPI, positive psychology intervention; TAU, treatment as usual. *P < 0.05.

Finally, we addressed the question of whether attitude to and/or evaluation of PPI resulted in a different disease outcome. We compared patients who did not value the treatment very much with those who did and those who had no experiences with PPI (TAU), on the basis of BDI-II levels (at admission and at discharge from hospital). Figure 3 demonstrates the results.

Our third hypothesis assumed that patients with positive evaluations of the PPI would have better overall treatment outcomes, and this was correct. As depicted in Figure 3, patients with positive attitudes toward PPI showed a higher reduction in BDI-II scores compared with patients with negative attitudes toward PPI, and they showed a significantly higher reduction in BDI-II scores compared with the control group (TAU). ANOVA with one repeated measurement factor (pre–post) clearly indicated a significant group by pre–post interaction (*F* = 3.20, d.f. = 2.71, *P* < 0.05, *η*^2^ = 0.09). Given that the difference in BDI-II scores is influenced by any treatment (including pharmacotherapy), it is noteworthy that nearly 10% of the variance can be explained. The highest reduction in BDI-II scores (indicating best therapy outcome) can be observed in patients with positive attitudes toward PPI. As indicated by Bonferroni-corrected *post hoc* tests with difference values (Fig. 3, right panel), this group significantly differed from TAU, whereas those with less positive attitudes were in between.

## Discussion

The present study investigated the effects of a 4-week PPI programme in the treatment of in-patients with depression. It administered treatment in a randomised fashion, and compared results against a control group that received TAU. Results indicate that a PPI is an effective extension in the treatment of patients with depression in an in-patient psychiatric setting, since joylessness is one of the key symptoms of depression. By controlling for patients’ comorbidities and their attitudes toward the treatment, the study has uncovered previously unknown clinical potential for PPIs.

Based on the literature, we postulated three hypotheses, which were supported by our findings. We hypothesised that positive effects of PPIs generalise to a sample of in-patients with severe depression, a larger number of comorbidities would dilute the beneficial effects of the PPI and beneficial effects of the PPI would be more pronounced in patients with positive attitudes toward the treatment.

Since the potential of PPIs had not been tested in an in-patient setting, the study's first hypothesis aimed at testing if it generalises to this population. Indeed, our patients displayed more positive and less negative emotionality after PPI sessions, compared with baseline. This change can be interpreted as responsiveness to the intervention, which is a precondition for treatment effectiveness. The fact that we found significant changes in affect (and therefore responsiveness) in a sample of in-patients with heavy psychological burden strongly supports the use of PPIs in this domain. However, the change in affect interacted with session number (1–4). Unlike in the first three sessions, there was no significant increase in positive affect in the very last session, and there was no significant reduction in negative affect in the final two sessions. A possible interpretation of this dampened responsiveness in the final session could be that some participants were aware of the fact that the fourth session was the last one, and after intensively working together for 3 weeks and given the overall positive evaluation of the PPI, felt pain at parting. Moreover, since the end of our PPI treatment often coincided with the end of our patients’ in-patient treatment, the final PPI session marked the beginning of this transition phase, which is a critical time in any psychotherapy. In this transition, patients have to cope with loss and complex emotional reactions, such as anger, anxiety and the re-emergence of symptoms.^26^

Our second hypothesis postulated that the effectiveness of PPI would be higher in patients with fewer comorbidities, and our results corroborated this assumption. To assess the effectiveness of the PPI treatment, we inspected the development of treatment-initial affect measures (i.e. the PANAS measures that were taken at the beginning of each session) over the course of the four PPI sessions. In patients with fewer comorbidities, we observed a significant increase in positive emotionality over time; in patients with more comorbidities, positive affect did not increase from week to week. This pattern is in line with previous research that showed an association of comorbidity with episode duration,^13,14^ and inferior therapeutic results in the treatment of patients with depression with comorbidities.^12^

Although our findings corroborate the hypothesis, we observed a difference between the changes in positive and negative affect that has not yet been described. Unexpectedly, the increase in positive affect was not mirrored by a decrease in negative affect. Although negative affect decreased slightly (see Fig. 2), the change was not significant and did not vary by number of comorbidities. In other words, negative affect measures remained stable. Thus far, the literature has mostly described increases in positive affect paralleled by decreases in negative affect.^4,5^ We see several possible explanations for our findings, which include differences in operationalisation of effectiveness, the independence of affect measures and a relatively short treatment duration. First, our measure of treatment effectiveness is less complex compared with other studies. Whereas other studies often used constructs such as subjective and psychological well-being (for example, the study by Sin and Lyubomirsky^4^), we focused on positive and negative affect as measured by the PANAS. Our decision to do so was motivated by a desire for simplicity, which lowered the burden on our patients and made the measure independent of cognitive evaluations (such as evaluations of one's general satisfaction or life purpose) present in more complex measures, and because we also considered BDI-II scores. Having both PANAS and BDI-II, state and trait components of emotionality can better be separated. Second, the fact that we see changes specific to positive affect might be because positive and negative affect (as measured by the PANAS) are independent of each other,^27^ and PPIs are specifically designed to target positive affect (recall that PPIs are constructed to cultivate positive feelings, behaviours or cognitions^4^). Another possible explanation is that because of our negativity bias, negative emotionality lasts longer and is more resistant toward change. Since the negativity bias is more pronounced in patients with depression,^28^ it is possible that PPIs are not sufficient to overcome negativity bias in samples with patients with severe depression. Finally, it might be that our 4-week treatment was too short to influence negative affect as longer PPIs produce larger changes in well-being.^4,5^

The third hypothesis postulated that patients with positive attitudes toward PPI would benefit more. Indeed, we found that patients who gave positive feedback after their first PPI session showed a higher reduction in BDI-II scores compared with those who were less enthusiastic and those in the TAU group. It can be concluded that PPI treatment can be especially effective if it takes patients’ individual preferences into account.

Attitude toward PPI turned out to be a good predictor of the efficacy of PPI. Therefore, patients’ characteristics are important for therapeutic outcomes. Since the identification of these aspects turned out to be extremely difficult because of statistical reasons (low power^29^), personalised psychotherapy was extremely difficult to achieve. However, we believe that the evaluation of therapeutic regimes should be included in every therapeutic process. If the first session of a specific programme turns out to be negatively evaluated by a patient, an alternative programme could be more beneficial compared with a rigid continuation based on the assumption that the patient does not respond because of their illness. Switching medication is very common after perceiving side-effects of low effectiveness; the same flexibility can be helpful for psychotherapeutic interventions as well.

Although the present study adds to the current knowledge on PPI in patients with depression, a few limitations must be noted. First, from the initial 54 patients in the PPI group, we could only use complete data-sets for 38 patients. However, this does not represent selective drop-out. The main reason was that participants were discharged from the hospital (e.g. when their therapy was successful) before they could complete the PPI programme (*n* = 9). Three other patients missed a PPI session because they had other important appointments (e.g. with child protective services), another two participants left the clinic prematurely because of low treatment adherence, one patient could not attend a single PPI session because of an acute psychiatric crisis with self-injuring behaviour and, finally, one patient had severe social phobia and could not tolerate the group setting. This demonstrates that our results are not affected by selective drop-out, and that missing values are entirely at random. However, the resulting reduction in sample size reduced statistical power in our sample.

Furthermore, it must be noted that a clinical trial like the one in our study always suffers from unstandardised elements. For example, there is variability in prescriptions, dosages of medications, drug profiles and polypharmacy. Changes in medication are frequent throughout therapies, which induces variance in clinical measures. Moreover, specific factors that can potentially confound our results should also be mentioned. In fact, we have one systematic difference between the PPI and the TAU group, which is the number of therapeutic interventions. As mentioned above, PPI treatment was applied in addition to all other interventions (including pharmacotherapy). This resulted in a difference of the total therapeutic interventions between the PPI and TAU groups. We faced this problem at the beginning of our study. An alternative could be to omit parts of the therapy in the PPI groups to achieve an identical amount of therapeutic interventions. However, we decided to accept this difference since omitting elements that are expected to be helpful and known to be beneficial raises ethical considerations. However, we believe that the shown effects within the PPI group (number of comorbidities, evaluation of PPI) are more significant than those demonstrated between TAU and PPI. In addition to these limitations, it should be acknowledged that our approach was conservative in that sense that we included in-patients with severe depression, which has rarely been done before.^6,8^

Further research should continue to illuminate the causes of successful PPIs to delineate the boundary conditions within which PPIs are most effective. A better understanding of these causes will pave the way toward personalised medicine that can improve therapeutic outcomes throughout. A promising starting point seems to be the investigation of causes of patients’ attitudes toward PPIs, which turned out to be a powerful predictor of the successful application of PPIs in the treatment of in-patients with severe depression. By restricting attention to the confounds measured here (age, gender, comorbidities, number of in-patient treatments and BDI-II scores), the causes of attitudes toward PPIs remain an enigma. Apart from attitudes toward PPIs, it also seems promising to follow current trends in medicine that increasingly consider genetic variation in the design of individual treatments.^30^ Certain genetic predispositions might shed new light on patients’ receptiveness of PPIs and other treatment methods.

## Data Availability

The data that support the findings of this study are available from the final author, J.H., upon reasonable request.
